# Application of AI Models for Preventing Surgical Complications: Scoping Review of Clinical Readiness and Barriers to Implementation

**DOI:** 10.2196/75064

**Published:** 2026-02-17

**Authors:** Kjersti Mevik, Ashenafi Zebene Woldaregay, Eva Lindell Jonsson, Miguel Tejedor, Claire Temple-Oberle

**Affiliations:** 1 Department of Breast and Endocrine Surgery, Nordland Hospital Trust, Bodø, 8092, Norway, 47 97123977; 2 Cumming School of Medicine, University of Calgary, Calgary, AB, Canada; 3 Department of Clinical Medicine, UiT The Arctic University of Norway, Tromsø, Norway; 4 The Norwegian Centre for Clinical Artificial Intelligence, University Hospital of North Norway, Tromsø, Norway; 5 Departments of Surgery and Oncology, Section of Plastic and Reconstructive Surgery, University of Calgary, Calgary, AB, Canada; 6 Department of Surgical Sciences, Plastic and Maxillofacial Surgery, Uppsala University, Uppsala, Sweden; 7 Technology and AI, Norwegian Centre for E-health Research, Tromsø, Norway; 8 Department of Mathematics and Statistics, UiT The Arctic University of Norway, Tromsø, Norway

**Keywords:** surgical complications prediction models, machine learning, artificial intelligence, AI, surgical complications, predictive modeling, risk prediction, surgery outcomes, perioperative care, clinical decision support

## Abstract

**Background:**

The impact of surgical complications is substantial and multifaceted, affecting patients and their families, surgeons, and health care systems. Despite the remarkable progress in artificial intelligence (AI), there remains a notable gap in the prospective implementation of AI models in surgery that use real-time data to support decision-making and enable proactive intervention to reduce the risk of surgical complications.

**Objective:**

This scoping review aims to assess and analyze the adoption and use of AI models for preventing surgical complications. Furthermore, this review aims to identify barriers and facilitators for implementation at the bedside.

**Methods:**

Following PRISMA-ScR (Preferred Reporting Items for Systematic Reviews and Meta-Analyses Extension for Scoping Reviews) guidelines, we conducted a literature search using IEEE Xplore, Scopus, Web of Science, MEDLINE, ProQuest, PubMed, ABI, Embase, Epistemonikos, CINAHL, and Cochrane registries. The inclusion criteria included empirical, peer-reviewed studies published in English between January 2013 and January 2025, involving AI models for preventing surgical complications (surgical site infections, and heart and lung complications or stroke) in real-world settings. Exclusions included retrospective algorithm-only validations, nonempirical research (eg, editorials or protocols), and non-English studies. Study characteristics and AI model development details were extracted, along with performance statistics (eg, sensitivity and area under the receiver operating characteristic curve). We then used thematic analysis to synthesize findings related to AI models, prediction outputs, and validation methods. Studies were grouped into three main themes: (1) duration of hypotension, (2) risk for complications, and (3) decision support tool.

**Results:**

Of the 275 identified records, 19 were included. The included models frequently demonstrated strong technical accuracy with high sensitivity and area under the receiver operating characteristic curve, particularly among studies evaluating decision support tools. However, only a few models were adopted routinely in clinical practice. Two studies evaluated the clinicians’ perceptions regarding the use of AI models, reporting predominantly positive assessments of their usefulness.

**Conclusions:**

Overall, AI models hold potential to predict and prevent surgical complications as the validation studies demonstrated high accuracy. However, implementation in routine practice remains limited by usability barriers, workflow misalignment, trust concerns, and financial and ethical constraints. The evidence included in this scoping review was limited by the heterogeneity in study design and the predominance of small-scale feasibility studies, particularly for hypotension prediction. Future research should prioritize prospectively validated models that use other physiologic features and address clinicians’ concerns regarding generalizability and adoption.

## Introduction

With more than 320 million surgical procedures performed worldwide annually, there is a global responsibility to enhance the quality of surgical care [1]. Complications such as surgical site infections and stroke or heart and lung complications, whether minor or severe, often lead to reoperations, morbidity, and prolonged hospital stay [2,3]. Death following surgery approaches 5% and every tenth patient experiences preventable surgical complications [1]. Adverse events also impact surgeons as a second victim [4], and health care resource use rises notably [5]. The added cost of surgical complications ranges from US $3.5 to $10 billion yearly and is associated with an average increase in hospital stay of 11 days [6]. Despite attempts to optimize adherence to clinical pathways to reduce the frequency of surgical complications, complications persist [7,8].

Artificial intelligence (AI) is transforming the field of surgery, offering unprecedented advancements in precision, efficiency, and patient outcomes that could possibly reduce surgical complications [9]. For example, AI-assisted frame reviews in neuroscience demonstrate that AI can help both junior and senior clinicians perform better [10]. In educational platforms, AI is outpacing traditional coaching programs as demonstrated by a gallbladder surgery program [11]. By leveraging the power of machine learning, natural language processing, and computer vision, AI can enhance various aspects of surgical practice from preoperative planning to intraoperative guidance to reduce surgical complications [12].

During preoperative discussions, the surgeon and patient must weigh the benefits of surgery against the risks. AI can rapidly process large amounts of health data, unburdening health personnel and allowing them to better inform their patients [13]. These AI models analyze patterns and their relationship to determine complex combinations that can indicate the patient’s risk for surgical complications. There is a gap in studies focusing on the adoption and clinical validation of these AI models [14-21]. Existing literature is focused on the retrospective development of models aimed at preventing surgical complications [9]. There are several reasons why these models have not been widely adopted in actual clinical use, including the lack of validation, lack of supporting data, differences in culture and behavior, and organizational structure [22,23]. Most common problems are regulatory and ethical constraints given that AI in surgery is considered high risk, time-consuming, and expensive. Randomized controlled trials (RCTs) are generally needed, which do not exist [24]. Also, there is currently a gap in the literature regarding the evaluation of AI models in clinical practice. This review aims to provide an overview of the published studies. Previous reviews have focused exclusively on studies involving the development and validation of models conducted using retrospective data [25-27]. Our focus is to uncover AI models that have been prospectively tested with real-time data at the bedside and to pinpoint the barriers and facilitators to implementing these models for the prevention of surgical complications.

## Methods

The review was conducted in accordance with the PRISMA-ScR (Preferred Reporting Items for Systematic Reviews and Meta-Analyses Extension for Scoping Reviews) guidelines (Checklist 1) [28]. We used a scoping review protocol that addressed key concepts and types of evidence. The aim was to map the existing literature by systematically searching, selecting, and synthesizing current evidence on AI models designed to prevent surgical complications using real-time data [29]. In this review, our definition of AI models also encompasses traditional statistical models, such as the National Surgical Quality Improvement Program (NSQIP), as these were tested prospectively. We relied on the five-stage framework proposed by Pollock et al [30]: (1) developing the review objective, (2) applying the eligibility criteria, (3) selecting the articles, (4) extracting and analyzing the data, and (5) reporting the results. The inclusion criteria were implemented using the population-concept-context framework, where population represents the patients undergoing surgery, concept includes the use of AI models to prevent surgical complications, and context includes studies that are conducted pre-, peri-, and intraoperative with both real-time and retrospective data excluding retrospective validation studies without prospective evaluation. The research team comprised both surgeons and machine learning engineers. The primary research question was “Which AI models have been clinically tested for the prediction of surgical complications?” The secondary research question was “For AI models not yet implemented in routine clinical practice, what barriers hinder their implementation, and are there any models on the horizon that could readily be adopted? ” We included prospective, observational, and interventional peer-reviewed studies in English that included model development, validation, and implementation. A comprehensive search strategy was developed in collaboration with a medical librarian to identify peer-reviewed original studies. We searched the following databases from October 2024 to January 2025: Scopus, CINAHL, the Cochrane Library, PubMed, MEDLINE, Web of Science, Embase, Epistemonikos, and IEEE Xplore [28]. The search strategy combined controlled vocabulary (eg, Medical Subject Headings and Emtree) and natural language keywords using Boolean operators and truncation to capture variations in terminology. The specific search terms and keywords were defined following iterative literature searches and several rounds of discussion among the authors.

The search query was structured around 3 key concepts:

- AI Methodology: (“Artificial intelligence” OR “Machine learning” OR “AI tool*” OR “AI model*” OR “Validated algorithm*”);
- Function: (“Predict*” OR “Prediction tool*” OR “Prediction index” OR “Clinical decision support tool”);
- Outcome: (“Postoperative complication*” OR “Surgical adverse event*” OR “Adverse surgical outcome*”).

The search was restricted to articles published in English. Studies were included if they described prospective model validation or clinical implementation.

A reference list of selected articles was used to extract additional articles to get a complete overview of the field. Detailed information on the search strategy can be found in Multimedia Appendix 1. The librarian vetted the initial search, using Mendeley (version 2.129.0; Elsevier). Eligibility assessment and screening were independently conducted by the primary investigator (KM) and co-investigator (ELJ) based on the established inclusion and exclusion criteria. After the initial screening, a full-text assessment was carried out. Disagreements were arbitrated by a third reviewer (CT-O). The reporting quality of the included studies was assessed by using the TRIPOD (Transparent Reporting of a Multivariable Prediction Model for Individual Prognosis or Diagnosis) with the AI statement [31,32]. The checklist includes 22 items (27 for the AI statement) with the potential answer options: “yes,” “no,” and “not applicable.” The 2 reviewers assessed the included studies for compliance with the items described in the TRIPOD+AI checklist. Furthermore, the following information was extracted: bibliographic details, study design and setting, surgical specialty and procedure type, AI model and technical details, predicted outcome or complications, stage of implementation, validation, and reported barriers and facilitators to clinical implementation. Quantitative characteristics derived from the included studies were summarized using tables and figures. Thematic analysis was performed on qualitative data related to barriers and facilitators. For this scoping review, we developed an analytical categorization framework to systematically classify the included studies according to the primary applications of the AI models they used (Table 1). This framework served to structure the evidence by grouping studies into conceptually coherent domains, thereby facilitating a clearer understanding of the thematic focus, methodological approaches, and applied contexts represented across the studies.

**Table 1.** Thematic classification of 19 included studies in the scoping review.

Theme	Studies (N=19), n (%)	AI model
Duration of hypotension	11 (58)	HPI^a^
Risk for complications	4 (21)	POTTER^b^, Periop ORACLE^c^, MuscleSound, and PPC -score
Decision support tool	4 (21)	My Surgery Risk, ACS^e^ NSQIP^f^, SURPAS^g^, and MyRISK

a HPI: Hypotension Prediction Index.

b POTTER: Predictive Optimal Trees in Emergency Surgery Risk.

c ORACLE: Outcome Risk Assessment with Computer Learning Enhancement.

d PPC: postoperative pulmonary complications.

e ACS: American College of Surgeons.

f NSQIP: National Surgical Quality Improvement Program.

g SURPAS: Surgical Risk Preoperative Assessment System.

## Results

**Figure 1.**
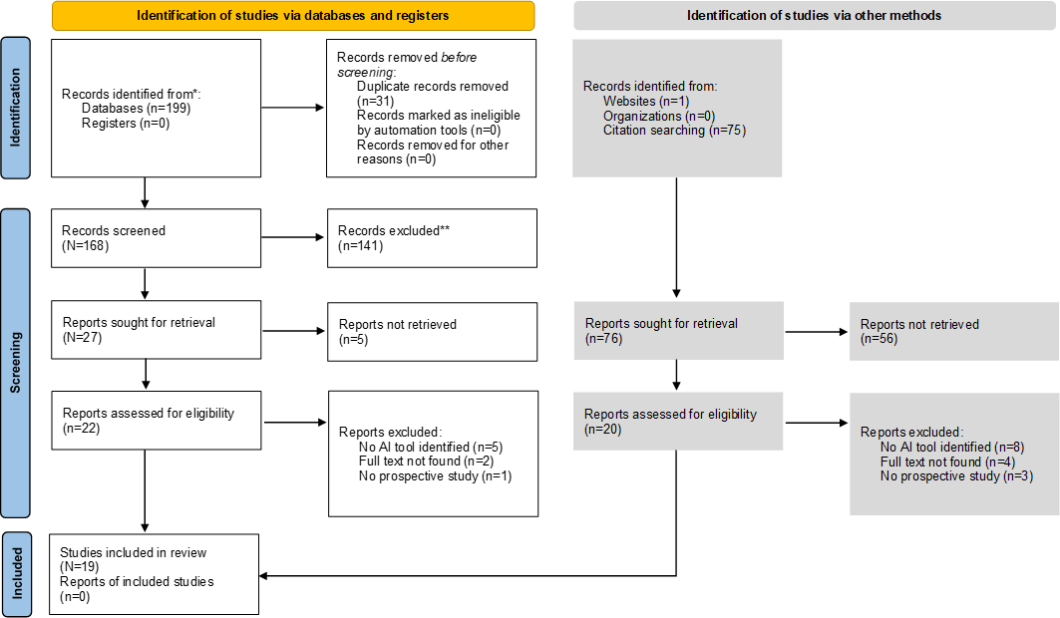
PRISMA-ScR (Preferred Reporting Items for Systematic Reviews and Meta-Analyses Extension for Scoping Reviews) flowchart (reproduced from Haddaway et al [34] and is covered by Creative Commons license). AI: artificial intelligence.

A PRISMA-ScR (Preferred Reporting Items for Systematic Reviews and Meta-Analyses Extension for Scoping Reviews) [33] flow diagram, as shown in Figure 1, illustrates the study selection process. The initial search yielded 199 articles, and 76 additional articles were gleaned from reference lists, for a total of 275 records screened. Of these 275 records, 19 studies met the inclusion criteria for this scoping review. The majority of studies were conducted in high-income countries, with the United States (n=7) being the most frequent contributor. The studies used a prospective study design, including RCTs, pilot interventional studies, and prospective cohorts. There were 8 RCTs and 11 prospective studies with a strong trend toward pilot-scale prospective studies. Large-scale validation or postdeployment studies were lacking. Few studies were evaluated outside of controlled research settings. External validation of the AI models was infrequent and adherence to TRIPOD+AI was poor. No study fully met the criteria for transparent reporting (Table 2).

**Table 2.** Summary of adherence to TRIPOD+AI (Transparent Reporting of a Multivariable Prediction Model for Individual Prognosis or Diagnosis + Artificial Intelligence) guidelines.

Study (year)	Model type	TRIPOD: outcome defined	TRIPOD: missing data	TRIPOD: internal validation	TRIPOD: external validation	TRIPOD‑AI: algorithm description	TRIPOD‑AI: explainability	TRIPOD‑AI: bias assessment
Wijnberge et al (2020) [35]	ML^a^-derived HPI^b^	✓		✓				
Lorente et al (2023) [36]	HPI protocol	✓		✓				
Schneck et al (2020) [37]	HPI system	✓		✓				
Bao et al (2024) [38]	Acumen HPI	✓	✓	✓				
Tsoumpa et al (2021) [39]	HPI algorithm	✓		✓				
Cylwik et al (2024) [40]	HPI software	✓		✓				
Murabito et al (2022) [41]	ML proactive HPI	✓		✓				
Šribar et al (2023) [42]	HPI-guided therapy	✓		✓				
Maheshwari et al (2020) [43]	ML-HPI tool	✓		✓				
Andrzejewska et al (2023) [44]	HPI prediction	✓		✓				
Kouz et al (2023) [45]	HPI registry	✓		✓				
Ren et al (2022) [46]	ML postoperative	✓	✓	✓		✓	✓	
Bilimoria et al (2013) [15]	ACS NSQIP Surgical Risk Calculator	✓	✓	✓	✓		✓	✓
Bronsert et al (2020) [47]	SURPAS^c^ pilot	✓	✓	✓			✓	✓
El Moheb et al (2023) [48]	Surgeon's AI risk	✓		✓		✓		
Ferré et al (2023) [49]	MyRISK score	✓	✓	✓		✓		
Fritz et al (2024) [50]	ORACLE^d^ ML model	✓	✓	✓		✓	✓	
Yik et al (2024) [51]	US sarcopenia AI	✓	✓	✓		✓	✓	✓
Li et al (2024) [52]	ML pulmonary outcome	✓	✓	✓		✓	✓	✓

a ML: Machine Learning.

b HPI: Hypotension Prediction Index.

c SURPAS: Surgical Risk Preoperative Assessment System.

d ORACLE: Outcome Risk Assessment with Computer Learning Enhancement.

To organize the heterogeneous literature, we developed a thematic categorization framework based on the primary intended function of each model as described in the original publications. The categorization was not based on the underlying statistical methodology, but on how the model output was framed and intended to be used in the clinical setting. Models categorized as “risk for complications” primarily focused on estimating the probability of specific postoperative outcomes, with performance evaluation as the main objective and limited emphasis on downstream clinical use. In contrast, models categorized as “decision support tools” were explicitly presented as supporting clinical decision-making processes, such as preoperative planning, shared decision-making, or patient counseling. We acknowledge that these categories are not mutually exclusive and that several models could reasonably fit more than one theme. In such cases, classification was based on the dominant emphasis in the study objectives and presentation (Table 1).

Study demographics and their respective funding are presented in Table 3. A total of 11 studies tested intraoperative hemodynamic monitoring and complication prediction using the Hypotension Prediction Index (HPI), while the remaining studies included AI models that addressed predicting general complications (n=7) and image analysis (n=1). While this review identified diversity of AI model applications, the majority of studies evaluated HPI, potentially skewing the findings through over-representation of intraoperative hemodynamic monitoring as the primary area of AI use in surgery. As such, the generalizability of results across surgical domains is narrowed. The predominance of HPI-related research may reflect a commercially available and well-integrated AI model, with greater funding and dissemination of pathways than other early-phase AI models. This may introduce publication and funding biases, where more rigorously tested, industry-supported models are over-represented compared to academic, noncommercial exploratory models. However, most of the studies testing the HPI had funding from the manufacturer Edwards Lifesciences. The authors stated that manufacturers were not involved in the conduct of the studies and did not approve or disapprove of the manuscript. Moreover, the clinical end points targeted by HPI are relatively narrow compared to the diverse risks associated with surgery. This limits the scope of AI use in surgery in terms of complication prediction, workflow optimization, and personalized surgical planning. Few studies evaluated AI models for long-term surgical complications, and the under-representation of other AI models narrows applicability. The main population studied was high-risk patients in 4 studies, with a subspecialty lens of noncardiac surgical patients. Table 4 delineated performance metrics of the AI models.

**Table 3.** Characteristics of the included articles.

Author (year) of publication	Country of origin	Clinical domain	Enrolled or planned participants	Type of study	Funding
Wijnberge et al (2020) [35]	The Netherlands	Noncardiac surgery	60	Unblinded randomized clinical trial: (1) early warning system with HPI^a^ and (2) standard care	Edwards Lifesciences
Lorente et al (2023) [36]	Spain	High-risk surgical patients for elective major abdominal surgery	80	Parallel-arm double-blinded multicenter randomized trial: (1) HPI protocol and (2) standard care	Edwards Lifesciences
Schneck et al (2020) [37]	Germany	Patients undergoing primary hip arthroplasty.	99	Single center randomized blinded prospective trial: (1) therapy algorithm HPI, (2) standard care, and (3) historic control group	Edwards Lifesciences
Bao et al (2024) [38]	The United States	Patients with ASA^b^ 3 or 4 moderate or high-risk noncardiac surgery >3 hours.	Prospective: 425 and post hoc analysis: 457 verss 15,796	Prospective single-arm multicenter (n=11) trial study: (1) continuous blood pressure measurements from study monitors compared to historical cohort with standard care study and (2) subset of trial participants versus a propensity score-weighted contemporaneous comparison group	Edwards Lifesciences
Tsoumpa et al (2021) [39]	Greece	Moderate or high-risk noncardiac surgery	99	Single-center prospective randomized trial: (1) HPI with hemodynamic treatment protocol and (2) standard care	None
Cylwik et al (2024) [40]	Poland	Patients undergoing oncological gastrointestinal surgery with ASA 3 or 4	46	Prospective single-center where HPI was used for 50 patients	None
Murabito et al (2022) [41]	Italy	Patients for elective major general surgery	40	Single-center pilot randomized clinical trial: (1) early warning system and (2) standard care	Edwards Lifesciences and the University of Catania
Šribar et al (2023) [42]	Croatia	Patients for elective major thoracic surgery with single lung ventilation	34	Prospective randomized single-center blinded trial: (1) “machine learning algorithm” (AcumenIQ) and (2) “conventional pulse contour analysis” (Flotrac)	None
Maheshwari et al (2020) [43]	The United States	Patients with ASA 3 or 4 for moderate or high-risk noncardiac surgery	214	Randomized multicenter controlled trial (n=2): (1) HPI guided group and (2) standard care	Edwards Lifesciences
Andrzejewska et al (2023) [44]	Poland	Patients undergoing posterior fusion for adolescent idiopathic scoliosis	59 adolescents	Prospective single-center, non-randomized, case-control study: (1) goal-directed therapy with HPI and (2) standard care	None
Ren et al (2022) [46]	The United States	Preoperative	67 surgeons testing the tool on 100 cases	Prospective	University of Florida, NIBIB^c^, NIDDK^d^, NIGMS^e^, and the National Science Foundation
Bilimoria et al (2013) [15]	The United States	Preoperative	80 surgeons testing the tool on 10 cases	Prospective	Agency for Healthcare Research and Quality
Bronsert et al (2020) [47]	The United States	Preoperative	197 patients assessed by 9 surgeons, but 166 were assessed by the tool	Convergent prospective mixed methods with both quantitative and qualitative data.	Agency for Healthcare Research and Quality
El Moheb et al (2023) [48]	The United States	Emergency surgery	150 patients, 15 surgeons in each group	Prospective, nonblinded, single-center: (1) prediction with use of POTTER^f^ and (2) standard prediction.	CRICO^g^ or RMF^h^ grant
Ferre et al (2023) [49]	France	Preoperative	389	Single-center prospective observational study	None
Kouz et al (2023) [45]	France, Germany, Italy, Spain, and the United Kingdom	Elective major noncardiac surgery	702	European multicenter (n=12) prospective observational trial	Edwards Lifescience
Fritz et al (2024) [50]	The United States	Patients for elective surgery during daytime weekdays	5071	Single-center prospective randomized clinical trial: (1) AlertWatch + ML^i^ display and (2) standard care	National Institute of Nursing Research, the Foundation for Anesthesia Education and Research, and the Washington University School of Medicine
Yik et al (2024) [51]	Singapore	Elective major gastrointestinal surgery	36	Prospective cohort study	SingHealth Medical Student Talent Development Award
Li et al (2024) [52]	China	Patient underwent surgical procedure with general anesthesia and mechanical ventilation	307	Prospective cohort in a single-center	The National Natural Science Foundation of China, Technology Project of Sichuan, Postdoctoral Science Foundation, Postdoctoral Program of Sichuan University, the Postdoctoral Program of West China Hospital, Sichuan, the 1$3$5 Project for Disciplines of excellence, West China Hospital, the Sichuan Province Natural Science Foundation of China, and the CAMS^j^ Innovation Fund for Medical Sciences

a HPI: Hypotension Prediction Index.

b ASA: American Society of Anesthesiologist Physical Status Classification System

c NIBIB: National Institutes of Health

d NIDDK: National Institute of Diabetes and Digestive and Kidney Diseases

e NIGMS: National Institute of General Medical Sciences

f POTTER: Predictive Optimal Trees in Emergency Surgery Risk.

g CRICO: Controlled Risk Insurance Company.

h RMF: Risk Management Foundation.

i ML: machine learning.

j CAMS: Chinese Academy of Medical Sciences

**Table 4.** Features and performance of the included artificial intelligence (AI) models.

Predicted outcome	Author (year) of publication	Features	Name of AI models	Performance metrics or clinical end points	Web-based calculators available
Duration of hypotension	Wijnberge et (2020) [35]	Arterial pressure waveform (28 variables)	HPI^a^	Median average hypotension: 0.10 mmHg versus 0.44 mmHg Median time of hypotension: 8 minutes and 33 minutes but no differences in adverse events The algorithm (HPI) tested with AUC=0.89 of 5-minute prediction time	Algorithm derivation only [53]
Duration of hypotension	Lorente et al (2023) [36]	Intraoperative time-weighted average of MAP^c^ <65 mm Hg, number of hypotension episodes, total time of hypotension, biomarkers of acute kidney distress, and tissue oxygenation	HPI	Median average hypotension: 0.06 mmHg versus 0 mmHg Median time of hypotension: 5 minutes and 0 minutes No differences in oxygen saturation and acute kidney injury	Algorithm derivation only [53]
Duration of hypotension	Schneck et al (2020) [37]	—	HPI	Duration of hypotension episodes: 0, 640, and 660 seconds	Algorithm derivation only [53]
Duration of hypotension	Bao et al (2024) [38]	Arterial pressure waveform and demographics, comorbidity, procedures, and acute kidney injury for post hoc analysis	HPI	58% reduction of MAP < 65 mmHg. In post hoc analysis, 35% reduction in minutes of hypotension Median time of hypotension: 9 minutes versus 15 minutes No difference in AKI^d^ 13.8% versus 15.8%	Algorithm derivation only [53]
Duration of hypotension	Tsoumpa et al (2021) [39]	Intraoperative time-weighted average of MAP <65 mm Hg, number and time of hypotension, amount of medicines, IV fluid, transfusion, morbidity, and complications	HPI	Median average hypotension: 0.16 mmHg and 0.50 mmHg Median time of hypotension: 9 minutes and 24 minutes No differences in complications or LOS^e^ or use of medicine or IV	Algorithm derivation only [53]
Duration of hypotension	Cylwik et al (2024) [40]	Pre- and postoperatively proBNP^f^ and troponin and acute kidney injury	HPI	Median average hypotension: 0.085 mmHg Median time of hypotension: 2 minutes Hypotension associated with acute kidney injury but not with myocardial injury	Algorithm derivation only [53]
Duration of hypotension	Murabito et al (2022) [41]	Time-weighted average of hypotension and biomarkers	HPI	Median average of hypotension: 0.12 mmHg and 0.37 mmHg Median time of hypotension: 4.3 minutes and 21.3 minutes Use of HPI reduced the intraoperative hypotension and biomarker for brain and oxidative stress	Algorithm derivation only [53]
Duration of hypotension	Šribar et al (2023) [42]	Time-weighted average of hypotension, intravenous fluids, medicines, ICU stay, length of stay, acute kidney injury, coronary syndrome, or cerebrovascular infarction	HPI	Median average of hypotension: 0.01 mmHg and 0.08 mmHg Median time of hypotension: 0 and 3.7 minutes	Algorithm derivation only [53]
Duration of hypotension	Maheshwari et al (2020) [43]	Arterial pressure waveform	HPI	Median average hypotension: 0.14 mmHg and 0.14 mmHg Median time of hypotension: 2 minutes and 2 minutes	Algorithm derivation only [53]
Duration of hypotension	Andrzejewska et al (2023) [44]	Surgical time, intravenous fluids, blood values, length of stay, and cardiac and neurological complications	HPI	Median hypotension time: 8 minutes and 40 minutes Less time to extubation time for the HPI group (median 5 vs 27.5 min) Noncardiac and neurological complications in the HPI group, while they were 4 in the control group	None
Duration of hypotension	Kouz et al (2023) [45]	Acute myocardial injury, acute kidney injury, death within 30 days after surgery, and hospital readmission within 30 days after surgery	HPI	Median time of hypotension: 2 minutes. Median average hypotension: 0.03 mm Hg 3% had acute myocardial injury, 9% had acute kidney injury Postoperative mortality within 30 days after surgery was observed in 2%	Algorithm derivation only [53]
Decision support tool	Ren et al (2022) [46]	285 inputs and 8 outcomes: complications and death	My Surgery Risk	Models with 135 features had AUC 0.80 to 0.92 for the different outcomes compared to lower AUC for models with 55 and 101 features Surgeon predictive performance did not change significantly after viewing predictions generated by the algorithm	Algorithm development [54,55]
Decision support tool	Bilimoria et al (2013) [15]	21 preoperative factors, 8 outcomes: mortality, morbidity, and 6 others	ACS^g^ NSQIP^h^ Surgical Risk Calculator	Brier score for mortality: 0.011 and morbidity: 0.069 Surgeons' agreement ranging from 80% to 100%	Algorithm development [56,57]
Decision support tool	Bronsert et al (2020) [47]	Mortality, overall morbidity, unplanned readmission, and 19 preoperative variables	SURPAS^i^	98.8% reported they understood their surgical risks very or quite well after exposure to SURPAS; 92.7% reported SURPAS was very helpful or helpful. Providers shared that 83.4% of the time they reported SURPAS was very or somewhat helpful; 44.7% of the time the providers reported it changed their interaction with the patient and this change was beneficial 94.3% of the time	None, but the algorithm can be found [58]
Risk for complication	El Moheb et al (2023) [48]	8 variables	POTTER^j^	POTTER outperformed surgeons in predicting mortality—AUC: 0.880 versus 0.841; ventilator dependence—AUC: 0.928 versus 0.833; bleeding—AUC: 0.832 versus 0.735; pneumonia—AUC: 0.837 versus 0.753	None, but the algorithm can be found [17]
Decision support tool	Ferre et al (2023) [49]	25 variables	MyRISK	AUC 0.71, sensitivity 94%, NPV^k^ 99%, specificity 49%, and PPV^l^ 7% Patient satisfaction 8/10 and usability 90/100	None, but validated in the same study.
Risk for complications	Fritz et al (2024) [50]	Variables within comorbidity, preoperative vital sign, preoperative laboratories, intraoperative time series, and medication and fluids	Periop ORACLE^m^	AUC for AKI: 0.73 and 0.69 Death: 0.79 and 0.78 No significant difference in prediction with the use of the model	None, but the algorithm can be found [59]
Risk for complications	Yik et al (2024) [51]	Intramuscular adipose tissue as a proxy for muscle quality obtained by ultrasound	MuscleSound	AUC 0.73 Clinicians using the tool can have a robust diagnostic tool to help predict surgical risk and outcomes	None
Risk for complications	Li et al (2024) [52]	20 variables	PPC^n^ score	AUC 0.88, simplified model AUC 0.86 Real-time identification of surgical patients' risk of postoperative pulmonary complications could help personalize intraoperative ventilatory strategies and reduce postoperative pulmonary complications	[60]

a HPI: Hypotension Prediction Index.

b AUC: area under the curve.

c MAP: mean arterial pressure

d AKI: acute kidney injury.

e LOS: length of stay.

f proBNP: pro-B-type natriuretic peptide.

g ACS: American College of Surgeons.

h NSQIP: National Surgical Quality Improvement Program.

i SURPAS: Surgical Risk Preoperative Assessment System.

j POTTER: Predictive Optimal Trees in Emergency Surgery Risk.

k NPV: negative predictive value.

l PPV: positive predictive value.

m ORACLE: Outcome Risk Assessment with Computer Learning Enhancement.

n PPC: postoperative pulmonary complications.

The American College of Surgeons (ACS) NSQIP Surgical Risk calculator is a widely adopted quality improvement tool used globally, and the HPI is a commercially available, regulatory-approved medical device (Acumen IQ) deployed in operating rooms. However, few of the other tools are deployed in surgical practice. In this regard, some of the common barriers included lack of external validation, limited generalizability, and black box model opacity (Table 5). Clinicians reported low trust in AI models (AI illiteracy and workflow issues as barriers) but noted real-time performance benefits (integration with existing platforms and clinical support as facilitators). Most of the AI models required high implementation costs, and together with the lack of financial incentives and reimbursement structures, these represented the greatest challenges to implementation.

**Table 5.** The most frequently cited barriers and facilitators in each of the studies.

Author (year of publication)	Barriers	Facilitators
Wijnberge et al (2020) [35]	Early warning system software needed (Flotrac IQ pressure transducer connected to the HemoSphere monitor)	No facilitator identified
Lorente et al (2023) [36]	Defining the correct range for normal blood pressure. FloTrac sensor (GDHT^a^ protocol) and AcumenIQ sensor (HPI^b^ protocol) needed.	No facilitator identified
Schneck et al (2020) [37]	Not mentioned	Implementation of HPI was considered uncomplicated providing a high user compliance
Bao et al (2024) [38]	FloTrac IQ sensor and EV1000 platform needed	No facilitator identified
Tsoumpa et al (2021) [39]	Acumen Flo-Traq transducer and EV1000 platform needed	No facilitator identified
Cylwik et al (2024) [40]	Need the HemoSphere monitoring platform, equipped with the AcumenTM IQ sensor	Anesthesiologists need training in the use of HPI software
Kouz et al (2023) [45]	Acumen IQ sensor (Edwards Lifesciences) and the HemoSphere monitoring platform are needed	Each center needs a clinical routine for hypotension procedures
Murabito et al (2022) [41]	FloTrac IQ sensor with EWS^c^ software needed	No facilitator identified
Šribar et al (2023) [42]	Hemosphere monitoring platform using either AcumenIQ or Flotrac sensors is needed	No facilitator identified
Maheshwari et al (2020) [43]	EV1000 is needed	The waveform needs to be acceptable using a fast flush test
Andrzejewska et al (2023) [44]	Acumen sensor and Hemosphere monitor are needed	Fast flush test was needed
Ren et al (2022) [46]	Fully automated data entry and mobile device outputs require a system architecture as a scalable real-time platform	Model outputs were provided to mobile device apps
Bilimoria et al (2013) [15]	Variables are manually added in the calculator	Allows clinicians to decrease the risk of surgery within the confidence interval for the predicted risk
Bronsert et al (2020) [47]	Variables are manually added in the calculator	Increase the interaction between the patient and the surgeon and make the patients able to understand the procedure and risk of the surgery.
El Moheb et al (2023) [48]	Variables are manually added in the calculator	Improved the surgeons' prediction
Ferre et al (2023) [49]	A digital questionnaire had to be filled out by the patients	The risks were visually illustrated with green (low), orange (intermediate), and red (high)
Fritz et al (2024) [50]	Variables are manually added in the calculator	No facilitator identified
Yik et al (2024) [51]	Need an ultrasound and the software Musclesound	Bedside and easy to use
Li et al (2024) [52]	Data developed on an older adult demographic	Easy to use and available online

a GDHT: goal-directed hemodynamic therapy

b HPI: Hypotension Prediction Index.

c EWS: early warning system

## Discussion

### Principal Findings

This scoping review highlights that only a small number of AI-based models have progressed to clinical use. Notably, ACS NSQIP is widely implemented as a quality improvement and risk stratification tool, whereas the HPI represents one of the few regulatory-approved AI-based medical devices that is ready for integration into routine clinical practice. The review identified several promising AI models that could help clinicians improve outcomes for surgical patients [61]. Although the models have demonstrated usefulness, important limitations remain regarding clinical use. Most of the studies reported a lack of widespread adoption. Although this review uses the umbrella term “AI-based models,” it is important to acknowledge the methodological heterogeneity of the included tools. Several widely used systems, such as ACS NSQIP, are based on traditional statistical approaches, primarily logistic regression, rather than modern machine-learning techniques. These models were included in accordance with our predefined search strategy, which intentionally captured both established statistical risk calculators and newer machine learning–based models used for surgical risk prediction and decision support. Importantly, traditional statistical models and machine learning algorithms differ in terms of model development, interpretability, data requirements, and generalizability. While logistic regression–based tools such as ACS NSQIP remain highly influential due to their transparency, validation history, and clinical acceptance, newer machine learning approaches offer potential advantages in handling complex, high-dimensional data but often face greater challenges related to interpretability, external validation, and clinical implementation. Distinguishing between these methodological paradigms is essential when interpreting the maturity and clinical readiness of AI-based tools in surgery.

Among the included models, HPI stood out as the most clinically mature and widely tested model, supported by multiple RCTs and integrated into well-known platforms. Its performance consistency and real-time application make it the most implementation-ready model in the surgical field. In contrast, the ACS NSQIP and ORACLE (Outcome Risk Assessment with Computer Learning Enhancement) demonstrated strong interpretability and user engagement but are designed exclusively for a preoperative decision support context, not intraoperative intervention. We observed that regulatory and ethical uncertainty is the most common reason why these models are not adopted into clinical practice. The lack of financial incentives to deploy AI is another barrier, elucidating why AI model development is progressing rapidly while translational science and implementation research lag behind [62]. A mixed method study suggested that barriers to implementing AI in clinical practice could be overcome by identifying and preparing champions, conducting educational meetings, promoting adaptability, and developing and disseminating educational materials on the AI model [63].

MySurgeryRisk, a tool developed by researchers at the University of Florida, uses machine learning to process vast amounts of patient data and clinical metrics and represents a promising predictive AI model with a high degree of accuracy. It is designed to provide real-time, actionable insights to surgeons, leading to better patient outcomes and optimized resource allocation [46,54]. A limitation of this model is that the predictions are mostly linear and do not account for combinations of variables that should be given greater weight when calculating risk. The augmentOR Portal developed by Asensus Surgical specifically evaluates the surgeon’s performance and identifies areas for improvement. This could reduce surgical complications by enhancing technical surgical skills but has yet to be trialed clinically [64]. The implementation of AI in predicting surgical complications is marked by these innovative approaches and promising results, yet its integration into routine clinical practice faces barriers [12,65].

Barriers that impede the widespread adoption of potentially transformative AI models in health care are several [66,67]. First, the quality and comprehensiveness of the data used to train these models are critical. AI models require extensive, well-annotated clinical data to learn effectively, and this data must be continually updated to reflect contemporary medical knowledge and practice [66]. Integrating AI models into existing health care IT infrastructures can be technically challenging and costly, necessitating significant upfront investment and ongoing maintenance, as well as extensive training of health care providers [68]. There are also substantial regulatory hurdles. AI models require rigorous testing and approval processes to ensure they meet clinical safety and efficacy standards. Ethical considerations, such as protecting patient privacy and avoiding biases in AI models, must be carefully managed to prevent disparities in health care outcomes [69]. For instance, the review by de Keijzer et al [70] highlighted that despite the potential of AI to transform clinical decision-making, there is a notable translational gap from proof-of-concept to clinical use. This gap is often due to regulatory uncertainties, organizational challenges, and attitudinal barriers among health care professionals. These barriers slow the uptake and adoption of AI models, even in cases where they have proved to significantly benefit patient care, such as in managing stroke complications [71]. Another reason for resistance to the implementation of AI is the skepticism of health care professionals towards AI models. Clinicians are cautious about relying on AI for decision-making, concerned that it may overlook individual patient nuances or erode their clinical autonomy [72,73]. Additionally, economic implications cannot be overlooked. The development, testing, and deployment of AI models require substantial financial resources, which can be a barrier for less well-funded health care institutions [74]. Finally, there is a worry that AI could become a substitute rather than a support for clinical decision-making, potentially leading to an erosion of clinicians’ professional skills [73]. Moreover, research exploring clinicians’ perceptions of AI underscores concerns regarding workload, risk, trust, and the integration of AI into clinical settings. Many clinicians fear that AI may increase their workload or change their workflow in ways that could compromise patient care. They also voice concerns about relying too heavily on technology that may not always account for the complex realities of medical practice [75]. Manual data entry is not feasible when the number of features is even moderately high. There would ideally be a bridge between AI models and the electronic health records, minimizing the effort for the clinicians to use the models. The integration should be a seamless solution, preferably as automated data pipelines that would facilitate implementations.

This review also reveals less explored but potentially transformative opportunities for advancing AI in surgery. For instance, embedding AI models into surgical training programs may foster early adoption and familiarity among new clinicians. Training curricula that include model interpretation and ethical consideration could empower the next generation of clinicians to embrace AI. Another often overlooked area is adaptive interface design. Many AI models fail because of poor integration into surgical workflows. Designing interfaces that adapt in real-time to the clinicians’ needs could make adoption more intuitive. Collaboration between data scientists and clinicians could advance in that direction. Moreover, the implementation of AI models in surgery still faces challenges in regulatory requirements, with a lack of alignment with existing clinical guidelines. A potential solution is the creation of sandbox-controlled environments where AI models can be evaluated and tested under close clinical and ethical oversight. Such frameworks, already explored in Fintech and digital health, could allow iterative deployment without compromising patient safety [76].

Regarding hypotension specifically, there have been several evaluations of HPI [77,78], including an RCT showing the efficacy of the HPI [70]. Retrospective studies have demonstrated that HPI reduces hypotension [79] which is associated with acute kidney injury and myocardial injury [80,81], and decreases mechanical ventilation time and length of intensive care unit stay [82]. To monitor the effect of HPI, a European registry has been established [83]. HPI is also used in a protocol to measure oxygen saturation and predict free flap survival [84]. HPI is ready for broader adoption, which may pave the way for more AI models in surgery. However, when it comes to decision support tools, once surgery is planned, the tool may not alter clinicians’ decisions due to the complexity of the inputs into existing prediction models. One concern with using AI for surgical decisions is the difficulty of integrating complex AI predictions into the nuanced and highly individualized process of surgical planning. AI models must accurately interpret and analyze medical images, the patient’s history, and other data to suggest surgical interventions. Currently, clinical judgment that is required for surgical decisions involves factors beyond what AI can predict, such as patient preferences, surgeon experience, and intraoperative findings. Thus, while AI can support decision-making and enhance specific tasks, it cannot replace the expert judgment of experienced surgeons [85].

### Conclusions

In conclusion, this scoping review demonstrates that despite substantial research activity, only a limited number of predictive models have been adopted into routine surgical practice. Most clinically implemented systems are based on traditional statistical models, such as ACS NSQIP, whereas only a few machine learning–based models, including the regulatory-approved HPI, have progressed toward clinical deployment. While these technologies show promise in improving perioperative risk prediction and physiological monitoring, current evidence does not consistently demonstrate downstream improvements in surgical outcomes. Continued technological advancements that can be deployed prospectively in controlled environments are important next steps. Such efforts are essential to safeguard patient safety, support the development of AI-specific reimbursement pathways within hospital budgets, and facilitate the integration of AI concepts into medical education to prepare future clinicians for AI-assisted clinical practice.

## Supplementary material

10.2196/75064 Multimedia Appendix 1 Search strategy.

10.2196/75064 Checklist 1 PRISMA-ScR checklist.

## Abbreviations

ACS: American College of Surgeons
AI: artificial intelligence
HPI: Hypotension Prediction Index
NSQIP: National Surgical Quality Improvement Program
ORACLE: Outcome Risk Assessment with Computer Learning Enhancement
PRISMA: Preferred Reporting Items for Systematic Reviews and Meta-Analyses
PRISMA-ScR: Preferred Reporting Items for Systematic Reviews and Meta-Analyses Extension for Scoping Reviews
RCT: randomized controlled trial
TRIPOD+AI: Transparent Reporting of a Multivariable Prediction Model for Individual Prognosis or Diagnosis + Artificial Intelligence
