# Supplementary material for: Application of AI Models for Preventing Surgical Complications: Scoping Review of Clinical Readiness and Barriers to Implementation
Source: JMIR AI. 2026 Feb 17;5:e75064. doi: 10.2196/75064 (PMC12912657; doi:10.2196/75064)
Supplement: Multimedia Appendix 1 [file ai-v5-e75064-s001.docx]

**Supplementary file 1: Search Strategy**

**Review Title:** Application of AI Models for Preventing Surgical Complications: A Scoping Review of Clinical Readiness and Barriers to Implementation

**Date of Search:** October 2024 to January 31, 2025

**Databases Searched:** PubMed, MEDLINE (Ovid), Embase (Ovid), Scopus, Web of Science, CINAHL, Cochrane Library, Epistemonikos, and IEEE Xplore.

**Overview of Search Logic** The search strategy was built on three core concepts based on the PICO framework:

1. **Index Test/Intervention:** Artificial Intelligence/Machine Learning
2. **Goal/Purpose:** Prediction or Clinical Decision Support
3. **Outcome:** Postoperative/Surgical Complications

Subject headings (MeSH for MEDLINE/PubMed/Cochrane, Emtree for Embase, CINAHL Headings) were exploded where appropriate and combined with keywords in the title and abstract fields.

**1. PubMed / MEDLINE**

**Interface:** National Library of Medicine **Date Searched**: 24 January 2025

| **Set** | **Search Query** |
| --- | --- |
| #1 | **Concept: Artificial Intelligence** ("Artificial Intelligence"[Mesh] OR "Machine Learning"[Mesh] OR "Deep Learning"[Mesh] OR "Neural Networks, Computer"[Mesh] OR "artificial intelligence"[Title/Abstract] OR "machine learning"[Title/Abstract] OR "AI tool*"[Title/Abstract] OR "AI model*"[Title/Abstract] OR "validated algorithm*"[Title/Abstract] OR "deep learning"[Title/Abstract]) |
| #2 | **Concept: Prediction & Support** ("Decision Support Techniques"[Mesh] OR "Prognosis"[Mesh] OR "Risk Assessment"[Mesh] OR "predict*"[Title/Abstract] OR "prediction tool*"[Title/Abstract] OR "prediction index"[Title/Abstract] OR "clinical decision support"[Title/Abstract] OR "risk stratification"[Title/Abstract]) |
| #3 | **Concept: Surgical Complications** ("Postoperative Complications"[Mesh] OR "Intraoperative Complications"[Mesh] OR "Surgical Procedures, Operative/adverse effects"[Mesh] OR "postoperative complication*"[Title/Abstract] OR "surgical adverse event*"[Title/Abstract] OR "adverse surgical outcome*"[Title/Abstract] OR "surgical mortality"[Title/Abstract]) |
| #4 | **#1 AND #2 AND #3** |
| #5 | Limit to English Language |

**2. Embase (Elsevier)**

**Interface:** Ovid **Date Searched:** 24 January 2025

| **Line** | **Search Query** |
| --- | --- |
| 1 | 'artificial intelligence'/exp OR 'machine learning'/exp OR 'deep learning'/exp OR 'neural network'/exp |
| 2 | 'artificial intelligence':ti,ab OR 'machine learning':ti,ab OR 'AI tool*':ti,ab OR 'AI model*':ti,ab OR 'validated algorithm*':ti,ab |
| 3 | #1 OR #2 |
| 4 | 'prognosis'/exp OR 'risk assessment'/exp OR 'decision support system'/exp |
| 5 | 'predict*':ti,ab OR 'prediction tool*':ti,ab OR 'prediction index':ti,ab OR 'clinical decision support':ti,ab |
| 6 | #4 OR #5 |
| 7 | 'postoperative complication'/exp OR 'peroperative complication'/exp OR 'surgical mortality'/exp |
| 8 | 'postoperative complication*':ti,ab OR 'surgical adverse event*':ti,ab OR 'adverse surgical outcome*':ti,ab |
| 9 | #7 OR #8 |
| 10 | **#3 AND #6 AND #9** |
| 11 | Limit to English Language |

**3. CINAHL (Cumulative Index to Nursing and Allied Health Literature)**

| **Line** | **Search Query** |
| --- | --- |
| S1 | (MH "Artificial Intelligence+") OR (MH "Machine Learning+") OR TI ("Artificial intelligence" OR "Machine learning" OR "AI tool*" OR "AI model*") |
| S2 | (MH "Decision Support Systems, Clinical+") OR (MH "Prognosis+") OR TI ("Predict*" OR "Prediction tool*" OR "Prediction index" OR "Clinical decision support") |
| S3 | (MH "Postoperative Complications+") OR TI ("Postoperative complication*" OR "Surgical adverse event*" OR "Adverse surgical outcome*") |
| S4 | **S1 AND S2 AND S3** |

**4. Scopus & Web of Science**

**Interface:** Elsevier / Clarivate Analytics *String adapted for wildcards and field codes.*

***(TITLE-ABS-KEY****("Artificial intelligence" OR "Machine learning" OR "AI tool*" OR "AI model*" OR "Deep learning" OR "Validated algorithm*")* ***AND*** ***TITLE-ABS-KEY****("Predict*" OR "Prediction tool*" OR "Prediction index" OR "Clinical decision support*" OR "Risk assessment")* ***AND*** ***TITLE-ABS-KEY****("Postoperative complication*" OR "Surgical adverse event*" OR "Adverse surgical outcome*" OR "Surgical complication*")****)*** ***AND LIMIT-TO****(LANGUAGE, "English")*

**5. Cochrane Library**

**Interface:** Wiley *Searched in: Cochrane Central Register of Controlled Trials (CENTRAL)*

| **ID** | **Search** |
| --- | --- |
| #1 | MeSH descriptor: [Artificial Intelligence] explode all trees |
| #2 | ("Artificial intelligence" OR "Machine learning" OR "AI tool" OR "AI model"):ti,ab,kw |
| #3 | #1 OR #2 |
| #4 | MeSH descriptor: [Postoperative Complications] explode all trees |
| #5 | ("postoperative complication" OR "surgical adverse event"):ti,ab,kw |
| #6 | #4 OR #5 |
| #7 | **#3 AND #6** |

**6. IEEE Xplore & Epistemonikos**

*Note: These databases utilize simplified Boolean logic and do not support full medical subject heading explosions.*

**Search String used:**

*("Artificial intelligence" OR "Machine learning" OR "AI model") AND ("Predict" OR "Prediction") AND ("Postoperative complication" OR "Surgical adverse event")*
